# Supplementary figures and images for: Liposomal Encapsulation of Polysaccharides (LEPS) as an Effective Vaccine Strategy to Protect Aged Hosts Against S. pneumoniae Infection
Source: Front Aging. 2021 Dec 22;2:798868. doi: 10.3389/fragi.2021.798868 (PMC8920316; doi:10.3389/fragi.2021.798868)

A.

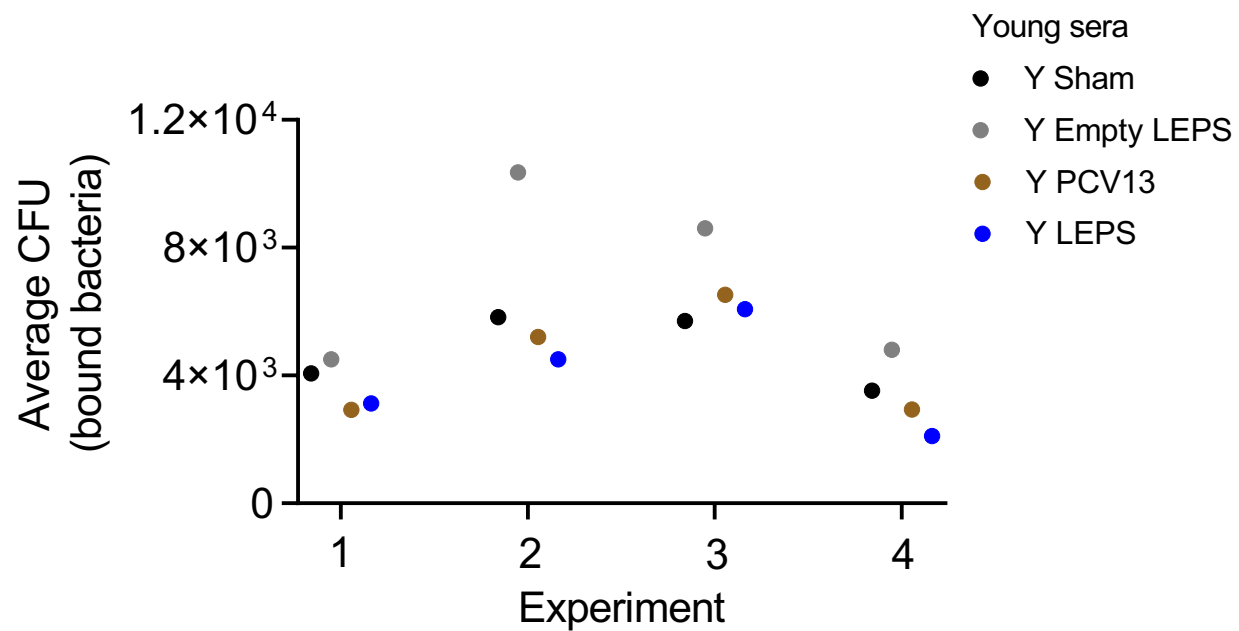

B.

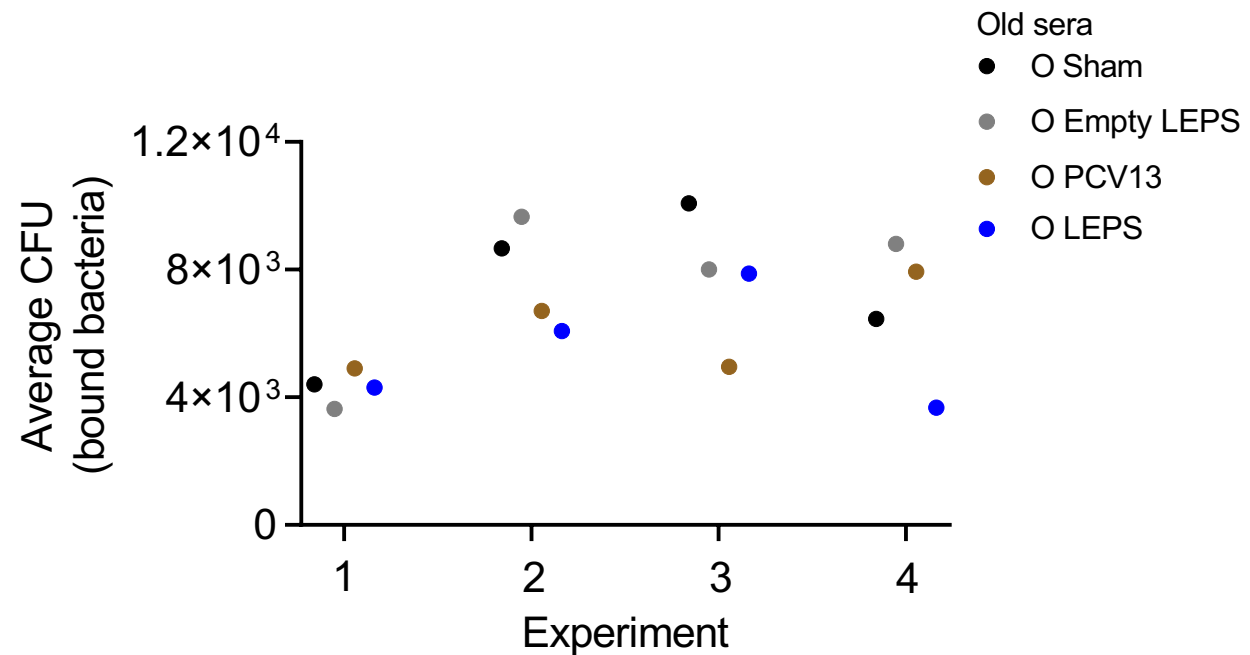

Supplement: Supplementary file 1 [file DataSheet2.PDF]

A.

IgM (anti-HK *Sp*)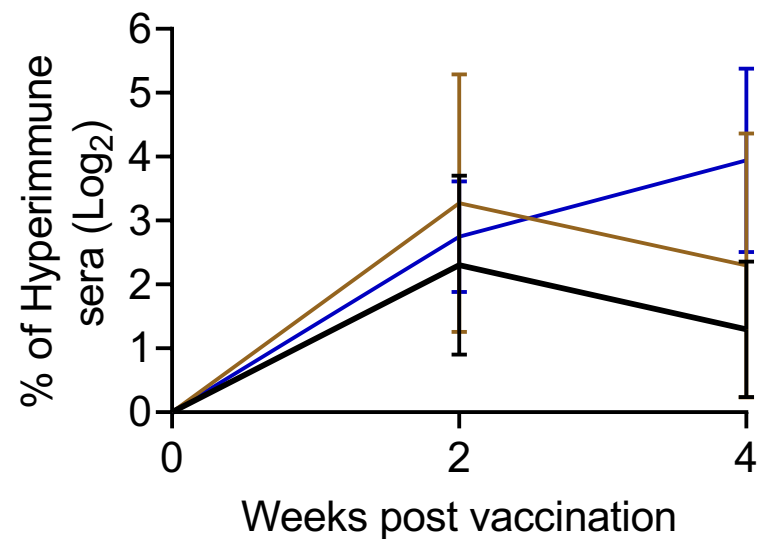

B.

IgG (anti-HK *Sp*)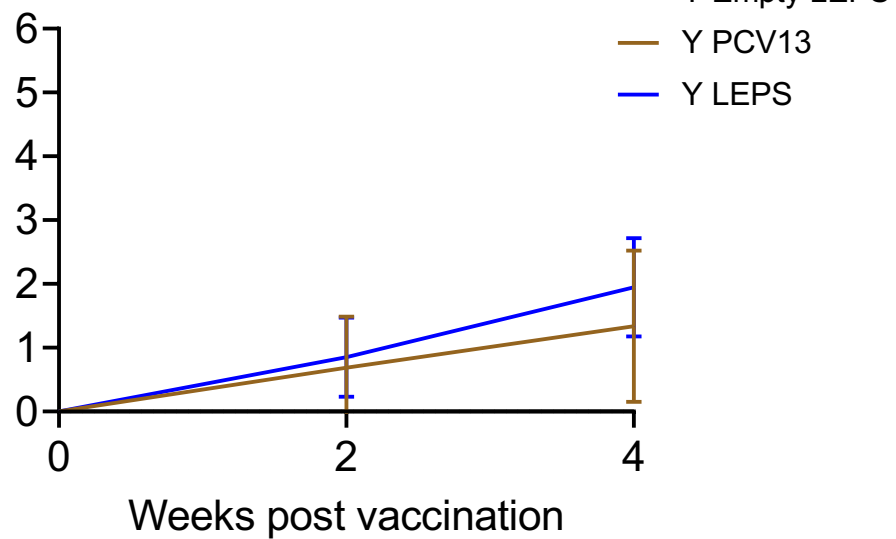

C.

IgM (anti-HK *Sp*)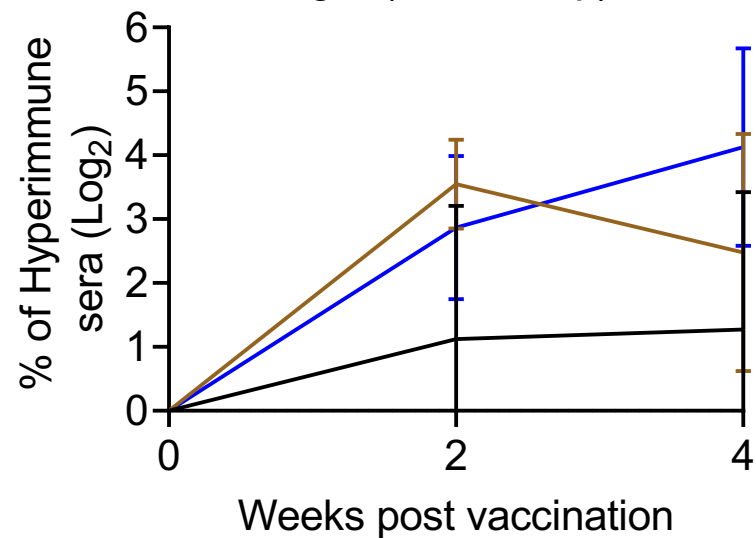

D.

IgG (anti-HK *Sp*)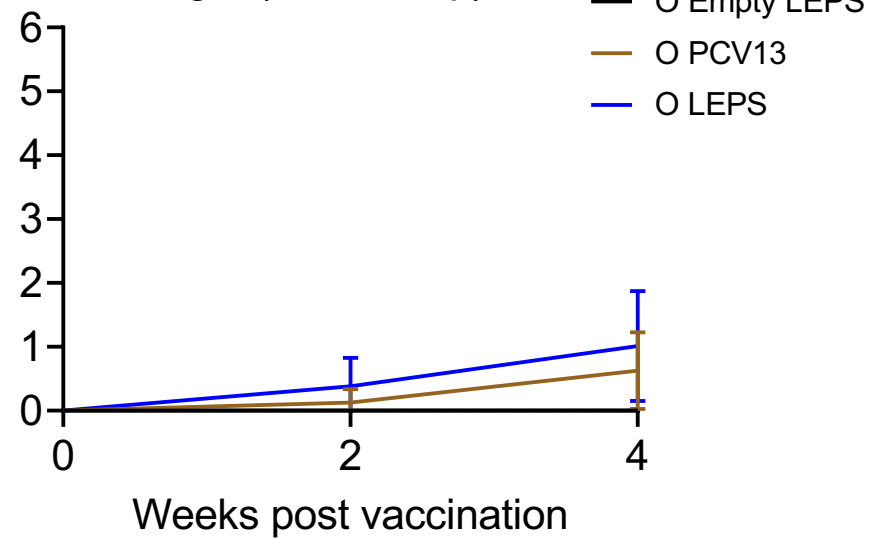

Supplement: Supplementary file 2 [file DataSheet1.PDF]
